# Supplementary material for: Andrographis Reverses Gemcitabine Resistance through Regulation of ERBB3 and Calcium Signaling Pathway in Pancreatic Ductal Adenocarcinoma
Source: Biomedicines. 2023 Jan 3;11(1):119. doi: 10.3390/biomedicines11010119 (PMC9855441; doi:10.3390/biomedicines11010119)
Supplement: Supplementary file 1 [file biomedicines-11-00119-s001.zip › biomedicines-2083277-supplementary.pdf]

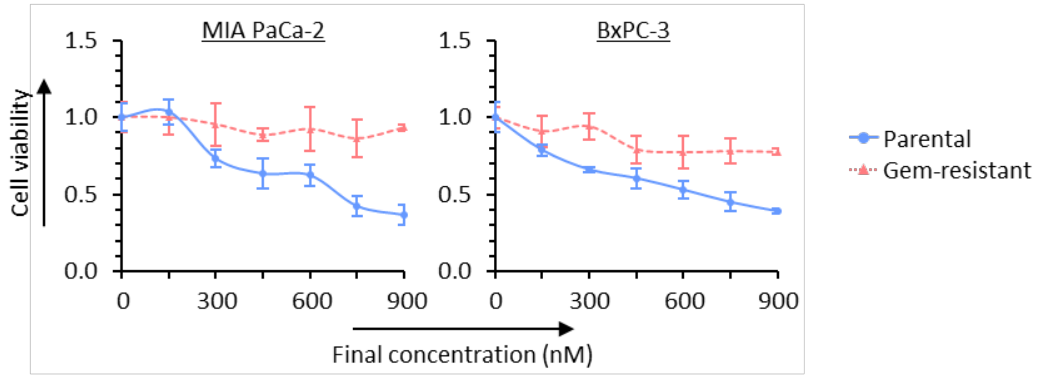

Supplementary figure S1: CCK-8 assays comparing cell viability following treatment with Gemcitabine for 48 hours in parental and Gem-R PDAC cells. Error bars are the mean ± SD. Gem-R, Gemcitabine-resistant; PDAC, pancreatic ductal adenocarcinoma.

Supplementary table S1: Patient characteristics of the TCGA dataset

	**TCGA** (n = 178)	
	n	(%)
Age, mean (±SD) (years)	65	(±11)
Sex		
Male	98	(55)
Female	80	(45)
Tumor location		
Head	139	(78)
Body	14	(8)
Tail	14	(8)
Unknown	11	(6)
Tumor size, mean (±SD) (mm)	39	(±17)
T stage		
1-2	31	(17)
3-4	145	(82)
Unknown	2	(1)
Lymph node metastases		
Negative	49	(27)
Positive	124	(70)
Unknown	5	(3)
Stage		
I-II	168	(94)
III-IV	8	(4)
Unknown	2	(1)

SD, standard deviation

Supplementary table S2: Patient characteristics of the clinical cohort

	Total (n = 37)	
	n	(%)
Age, mean (±SD) (years)	67	(±9)
Sex		
Male	21	(57)
Female	16	(43)
Tumor location		
Head	12	(32)
Body	20	(54)
Tail	5	(14)
Tumor size, mean (±SD) (mm)	43	(±15)
T stage		
3	14	(38)
4	23	(62)
Lymph node metastases		
Negative	12	(32)
Positive	25	(68)
Stage		
III	4	(11)
IV	33	(89)
Regimen		
Gem + nab-paclitaxel	35	(95)
Gem + S-1	2	(5)
RECIST 1.1		
PR	10	(27)
SD	13	(35)
PD	14	(38)
Responders or Non-responders		
Responders	23	(62)
Non-responders	14	(38)

SD, standard deviation; RECIST, response evaluation criteria in solid tumors; PR, partial response; SD, stable disease; PD, progressive disease; Gem, Gemcitabine; nab-paclitaxel, albumin-bound Paclitaxel; S-1, Tegafur /Gimeracil /Oteracil

Supplementary table S3: Primer sequences and their PCR conditions in this study

Gene	Sense	Antisense	Size of the PCR products (bp)	Annealing temp (°C)	GenBank accession No
ERBB3	GACCCAGGTCTACGATGGGAA	GTGAGCTGAGTCAAGCGGAG	99	60	NM_001982
Bcl-2	GGTGCCACCTGTGGTCCACCTG	CTTCACTTGTGGCCCAGATAGG	459	60	NM_000633
Cyclin D1	ACCTGGATGCTGGAGGTCT	GCTCCATTTGCAGCAGCTC	241	60	NM_053056
Caspase-3	CAAACTTTTTCAGAGGGGATCG	GCATACTGTTTCAGCATGGCAC	262	60	NM_032991
β-*actin*	AGAGCTACGAGCTGCCTGAC	AGCACTGTGTTGGCGTACAG	184	60	NM_001101
